# Cas9-Mediated Nanopore Sequencing Enables Precise Characterization of Structural Variants in *CCM* Genes

**DOI:** 10.3390/ijms232415639

**Published:** 2022-12-09

**Authors:** Dariush Skowronek, Robin A. Pilz, Loisa Bonde, Ole J. Schamuhn, Janne L. Feldmann, Sabine Hoffjan, Christiane D. Much, Ute Felbor, Matthias Rath

**Affiliations:** 1 Department of Human Genetics, University Medicine Greifswald and Interfaculty Institute of Genetics and Functional Genomics, University of Greifswald, 17475 Greifswald, Germany; 2 Department of Human Genetics, Ruhr-University, 44801 Bochum, Germany; 3 Department of Human Medicine and Institute for Molecular Medicine, MSH Medical School Hamburg, 20457 Hamburg, Germany

**Keywords:** nanopore sequencing, long-read sequencing, CRISPR/Cas9, copy number variants, cerebral cavernous malformations, structural variants

## Abstract

Deletions in the *CCM1*, *CCM2*, and *CCM3* genes are a common cause of familial cerebral cavernous malformations (CCMs). In current molecular genetic laboratories, targeted next-generation sequencing or multiplex ligation-dependent probe amplification are mostly used to identify copy number variants (CNVs). However, both techniques are limited in their ability to specify the breakpoints of CNVs and identify complex structural variants (SVs). To overcome these constraints, we established a targeted Cas9-mediated nanopore sequencing approach for CNV detection with single nucleotide resolution. Using a MinION device, we achieved complete coverage for the *CCM* genes and determined the exact size of CNVs in positive controls. Long-read sequencing for a *CCM1* and *CCM2* CNV revealed that the adjacent *ANKIB1* and *NACAD* genes were also partially or completely deleted. In addition, an interchromosomal insertion and an inversion in *CCM2* were reliably re-identified by long-read sequencing. The refinement of CNV breakpoints by long-read sequencing enabled fast and inexpensive PCR-based variant confirmation, which is highly desirable to reduce costs in subsequent family analyses. In conclusion, Cas9-mediated nanopore sequencing is a cost-effective and flexible tool for molecular genetic diagnostics which can be easily adapted to various target regions.

## 1. Introduction

Cerebral cavernous malformations (CCMs) are thin-walled vascular lesions in the central nervous system which are prone to hemorrhage. Depending on their location, they can cause a wide range of neurological complications, such as stroke-like symptoms or seizures. The familial form of CCM, which is characterized by the presence of multiple CCMs, follows an autosomal-dominant inheritance pattern with incomplete penetrance and is caused by heterozygous germline variants in the *CCM1 (KRIT1)*, *CCM2*, or *CCM3 (PDCD10)* gene [1].

Disease-causing variants are found in more than 90% of all familial cases [1]. Frameshift mutations account for the vast majority of pathogenic variants, followed by nonsense, splice site, and copy number variants (CNVs) [1,2,3,4]. Indeed, up to 18% of pathogenic variants found in CCM patients are large deletions [5]. Standard diagnostic techniques for CNV detection are multiplex ligation-dependent probe amplification (MLPA) and short-read gene panel sequencing in combination with specific bioinformatic pipelines. However, both methods have limitations. For example, they can hardly identify the precise breakpoints of CNVs, pathogenic variants in non-coding regions, and complex structural variants (SVs), e.g., inversions or interchromosomal insertions. This is particularly relevant since all these types of genetic variation have been described for CCM patients in the recent literature [6,7,8,9]. In the context of analyses for CNVs segregating within CCM families, cost-effectiveness is also an issue. 

Besides short-read-based methods, alternative approaches like Linked-Reads by 10x Genomics, Strand-Seq, or Optical Mapping have been successfully used for SV calling. Long-read based sequencing platforms like PacBio or Oxford Nanopore have also emerged as reliable tools for identifying SVs [10]. Long-read sequencing has the potential to overcome some limitations of current short-read sequencing-based molecular genetic diagnostics. It allows bridging and precise identification of SV breakpoints, making it a robust tool in CNV detection [11,12]. In particular, the highly flexible and yet affordable MinION platform from Oxford Nanopore, which enables read lengths of more than 30 kb [13], can be a valuable tool for almost any diagnostic lab. For targeted long-read sequencing, regions of interest can be enriched by using long-range PCR [14], hybridization-based approaches [15,16], Xdrop systems [17], adaptive sampling [18], or CRISPR/Cas9-mediated target selection, which enables amplification-free sequencing of genomic DNA [19] and has already been used successfully to identify SVs in clinically relevant genes like *BRCA1* [20]. 

Depending on the specific question and the available information from previous analyses, either a single- or dual-cut excision approach should be applied for CRISPR/Cas9-mediated nanopore sequencing. In the single-cut approach, a crRNA with a binding site near one end of the target region is used to read into the unknown one. This strategy is used for genome walking approaches or when only one side of the target region is known [21]. However, the coverage often drops toward the end of the target region. The dual-cut excision approach is based on the design of crRNAs at each end of the target region and results in higher coverage [21]. However, both ends of the target region need to be known. A combination of both approaches can be applied to larger regions [22].

In this study, we show that nanopore sequencing in combination with Cas9-mediated target selection can serve as an excellent complement to diagnostic short-read sequencing of the *CCM* genes. Detection of CNVs at single nucleotide resolution with moderate requirements for hardware and bioinformatics skills enables cost-effective and rapid PCR approaches for subsequent cascade analyses in CCM families. Furthermore, we demonstrate that even complex SVs can be reliably detected with targeted nanopore sequencing. 

## 2. Results 

### 2.1. Cas9-Mediated Nanopore Sequencing Confirmed a 2552 bp Deletion in CCM1

To cover the entire genomic loci of *CCM1*, *CCM2*, and *CCM3* with long reads, we first designed specific crRNAs to facilitate nanopore sequencing (Table S1). For each gene, the crRNAs were located approximately 15 kb apart from each other to avoid incomplete coverage of the target regions. Enrichment was performed in three separate reactions, allowing parallel sequencing of the three *CCM* genes in one sequencing run and ensuring that sequencing reads would not be terminated early by another CRISPR/Cas9-induced double-strand break. Whenever possible, crRNAs were designed to facilitate the same sequencing direction in a walking approach.

**Figure 1:**
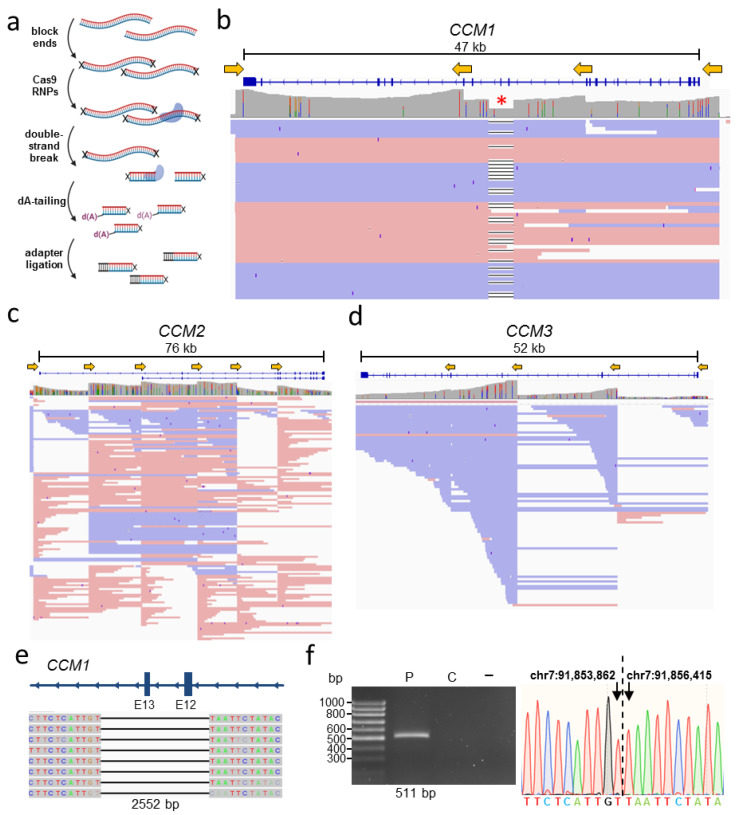
Cas9-targeted nanopore sequencing of the *CCM* genes precisely re-identified a two-exon deletion in *CCM1*. (**a**) Schematic representation of the sample preparation protocol used for Cas9-targeted nanopore sequencing. (**b**) *CCM1* sequencing data showed a two-exon deletion spanning from intron 11 to intron 13 (*, red star). CrRNA binding sites and sequencing orientation are symbolized by yellow arrows. The coverage with a peak at 127× is displayed in gray. An excerpt of the generated reads is shown in red and blue. (**c**,**d**) *CCM2* and *CCM3* long-read sequencing data. The highest coverage was 119× and 87× for *CCM2* and *CCM3*, respectively. The generated reads are shown in red and blue. (**e**) Breakpoints of the 2552 bp deletion in *CCM1* were visually inspected in the Integrated Genomics Viewer (IGV). (**f**) Deletion-specific PCR detected a 511 bp band in the proband sample (P) and its absence in a healthy control (C) and negative control sample (−). Sanger sequencing of the extracted band revealed the exact breakpoints. The genomic location is based on the GRCh37 reference genome. Read data (**b**–**e**) were visualized in IGV [23]. The Locus Reference Genomic (LRG) transcripts are shown (**b**–**e**).

To validate our new crRNA-panel, we sequenced a sample with a known two-exon deletion in *CCM1*. High-molecular-weight genomic DNA was extracted, and target selection was performed by Cas9-mediated induction of double-strand breaks and ligation of sequencing adapters to cleaved ends following the Oxford nanopore protocol (Figure 1a). Long-read sequencing successfully mapped the *CCM1* deletion, which started in intron 11 and ended in intron 13 with a mean sequencing coverage of 89.5× for *CCM1* (SD = 19.6; Figure 1b; Table S2). In the same run, sequencing of *CCM2* and *CCM3* confirmed the absence of other SVs in the sample while achieving a mean sequencing coverage of 71.9× (SD = 25.3) and 31.0× (SD = 18.9), respectively (Figure 1c,d; Table S2). To refine the CNV breakpoints, we used the cuteSV tool and the Sniffles2 structural variant caller, which is implemented in the EPI2ME Labs software (Table S4). Visual inspection of our long-read data in the Integrative Genomics Viewer (IGV) finally enabled the design of deletion-specific primers (Figure 1e). The 2552 bp deletion in *CCM1* and its exact breakpoints were validated by PCR and Sanger sequencing (Figure 1f; Table S5). 

Because the initial sequencing depth for some areas in *CCM2* and *CCM3* was relatively low, we revised our *CCM* crRNA-panel for the following sequencing runs. The final panel combined the walking approach with the dual-cut excision approach, in which two cuts are made, one upstream and one downstream of the target region, to generate optimal coverage for *CCM1*, *CCM2*, and *CCM3* (Table S1). 

### 2.2. Targeted Nanopore Sequencing Revealed the Exact Size of Large Deletions in Familial CCM Cases 

Current detection methods focus on intragenic CNVs in CCM patients. For this reason, the size of variants that are partially extragenic cannot be further characterized. Therefore, we wanted to test whether our long-read sequencing approach could specify variant breakpoints of large deletions spanning gene boundaries since this would allow the development of cost-effective PCR-based assays for familial analyses. We used a targeted approach with crRNAs located upstream of the first deleted exon that had been previously identified by NGS or MLPA analysis. 

**Figure 2:**
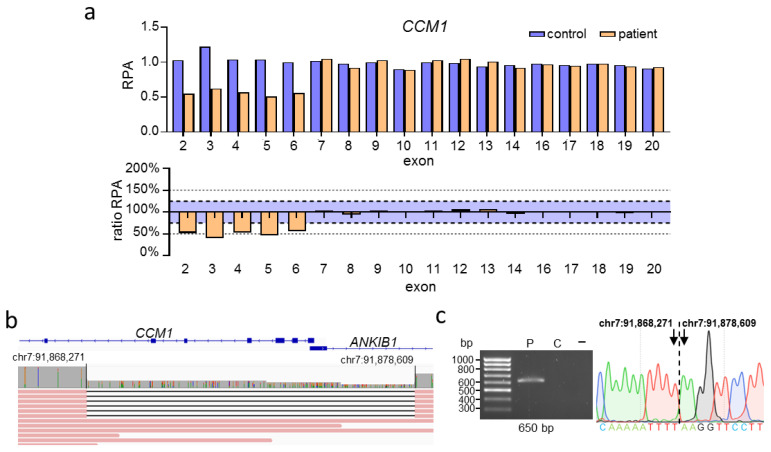
Nanopore sequencing of a large deletion in *CCM1* identified by MLPA revealed a partial deletion of *ANKIB1*. (**a**) MLPA revealed the heterozygous deletion of exons 2–6 of *CCM1* (RPA = relative product area). An exon is considered to be deleted or duplicated if the ratio RPA is below 75% or above 125%, respectively (blue area). (**b**) Subsequent nanopore sequencing showed that the deletion comprises 10,337 base pairs, spanning from *CCM1* intron 6 to *ANKIB1* intron 1. Read data were inspected in IGV [23]. (**c**) Deletion-specific PCR detected a 650 bp band in the sample of the index proband (P) and its absence in a healthy control (C) and negative control sample (−). Sanger sequencing of the extracted band revealed the exact deletion breakpoints. The genomic location is based on the GRCh37 reference genome. The Locus Reference Genomic (LRG) transcript (*CCM1*) and RefSeq transcript (*ANKIB1*) are shown (**b**).

CNV analysis by MLPA for the proband presented in Figure 2 identified a heterozygous deletion of the *CCM1* exons 2 to 6 (Figure 2a). No data could be generated for the noncoding exon 1 of *CCM1* because the commercially available MLPA kit did not contain specific probes for this region. Over the next three years, nine relatives were tested for the presence of the variant using MLPA. We reanalyzed the DNA of the index proband by nanopore sequencing (Table S3), which revealed the deletion to be larger than anticipated. The deletion actually spans from intron 1 of the adjacent *ANKIB1* gene to intron 6 of *CCM1* (Figure 2b; Table S4). These results were confirmed by deletion-specific PCR and Sanger sequencing (Figure 2c; Table S5). Long-read sequencing not only enabled the design of a deletion-specific PCR assay, which can be used for further familial analyses, but also demonstrated partial deletion of *ANKIB1*. So far, however, little is known about the function of ANKIB1. 

**Figure 3:**
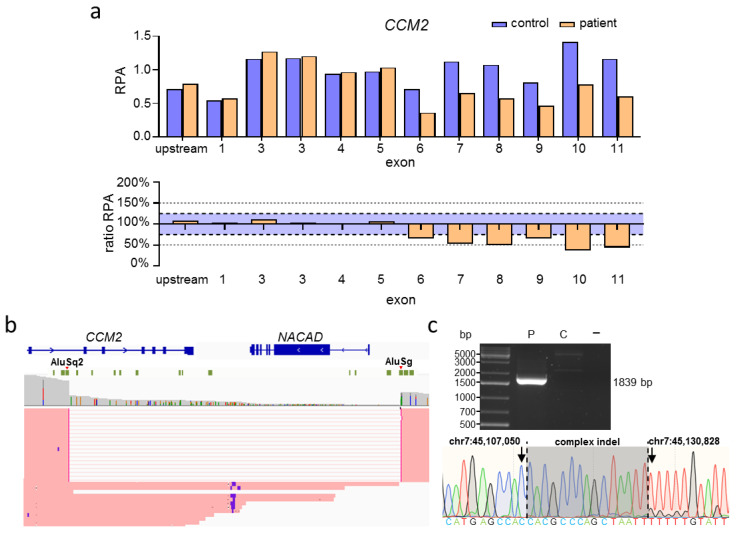
Nanopore sequencing of a large *CCM2* deletion suggests an Alu-mediated origin. (**a**) MLPA revealed a heterozygous deletion of exons 6–11 of *CCM2* (RPA = relative product area). An exon is considered to be deleted or duplicated if the ratio RPA is below 75% or above 125%, respectively (blue area). (**b**) Subsequent nanopore sequencing showed that the deletion comprises 23,777 bp, starting in intron 5 of *CCM2* and encompassing the neighboring gene *NACAD*. Deletion breakpoints are located inside of an AluSq2 and an AluSg repeat, respectively (green blocks). Read data were inspected in IGV [23]. (**c**) Deletion-specific PCR detected a 1839 bp band in the sample of the proband (P) and its absence in a healthy control (C) and negative control sample (−). Sanger sequencing of the extracted band was able to specify the deletion breakpoints and revealed a 15 bp indel variant. The genomic location is based on the GRCh37 reference genome. The Locus Reference Genomic (LRG) transcript (*CCM2*) and RefSeq transcript (*NACAD*) are shown (**b**).

For the proband presented in Figure 3, CNV analysis by MLPA was able to identify a multi-exon deletion in *CCM2*, spanning from exon 6 to exon 11 (Figure 3a). Nanopore sequencing (Table S3) revealed the true size of the deletion to be 23,777 bp, covering the complete *NACAD* gene located downstream of *CCM2* (Figure 3b). So far, the function of NACAD is unknown. Visual inspection in IGV suggested that this deletion originated from Alu-mediated recombination (Figure 3b; Table S4). Since breakpoints were localized in these highly repetitive regions, sequence alignment was not able to confidently determine the exact distal breakpoint. Nevertheless, we were able to design specific PCR primers to confirm the deletion by PCR and identify the exact breakpoints by Sanger sequencing, which also revealed an additional 15 bp indel variant between the breakpoints (Figure 3c; Table S5). 

Unfortunately, we were not able to apply our approach to re-analyze CNVs within *CCM3* since no appropriate samples were available.

### 2.3. Targeted Nanopore Sequencing Can Be Used to Identify Complex SVs in CCM 

**Figure 4:**
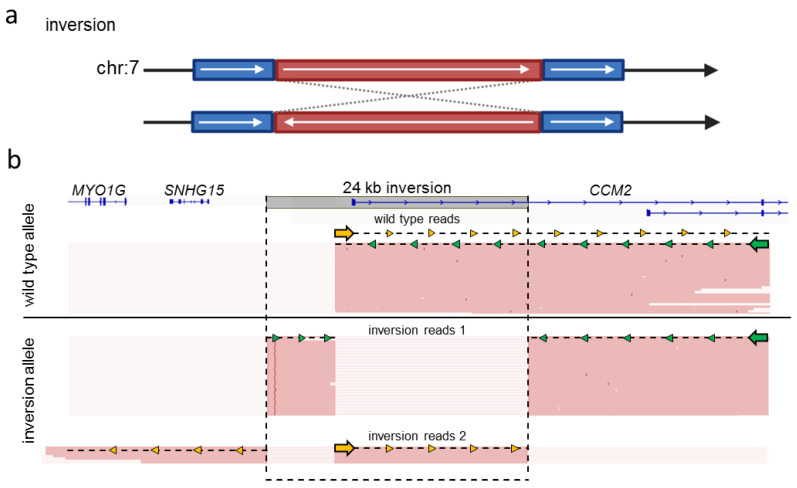
Nanopore sequencing confidently detected a 24 kb inversion in *CCM2*. (**a**) Schematic representation of the inversion in *CCM2*. (**b**) A dual-cut approach was used to re-sequence a sample with a known heterozygous 24 kb inversion in *CCM2.* CrRNAs facilitated sequencing in opposing directions. One binding site was located inside of the inversion (yellow arrow), and the other one was located downstream of the variant (green arrow). Sequencing of the wild-type allele resulted in one type of reads localized between both crRNA cut sides. Two distinct read patterns visualizing the inversion could be observed depending on which crRNA initiated sequencing of the inversion allele (inversion reads 1; inversion reads 2). Read data were inspected in IGV [23]. The Locus Reference Genomic (LRG) transcripts are shown (**b**).

The detection of complex SVs is hard or even impossible with short-read gene panel sequencing. Since the identification of an interchromosomal insertion and an inversion in *CCM2* [8,9] suggests that SVs must be considered as a possible cause of familial CCM disease, we wanted to test whether long-read sequencing could accurately detect these variants. Therefore, we reanalyzed a heterozygous 24 kb inversion on chromosome 7 (Figure 4a), which covers the first coding exon of *CCM2* [8], with long-read sequencing (Figure 4b; Table S3). We used crRNAs that were part of our *CCM* crRNA-panel to facilitate a dual-cut excision approach covering roughly half of the coding region of *CCM2*. The crRNA located upstream of *CCM2* was within the variant boundaries of the 24 kb inverted region, whereas the downstream crRNA was located in *CCM2* intron 3. The wild-type allele was present in roughly half of all reads. Two different kinds of reads covering the inversion were present. In both cases, mapping orientation flipped after passing one of the inversion breakpoints (Figure 4b). Due to the different types of reads generated by sequencing the inversion allele, we were able to identify both breakpoints of the 24 kb inversion. This variant was discovered previously only using short-read WGS [8]. As the variant is copy number neutral and the breakpoints lie in non-coding regions, detection via targeted panel sequencing, whole-exome sequencing, or MLPA was impossible.

**Figure 5:**
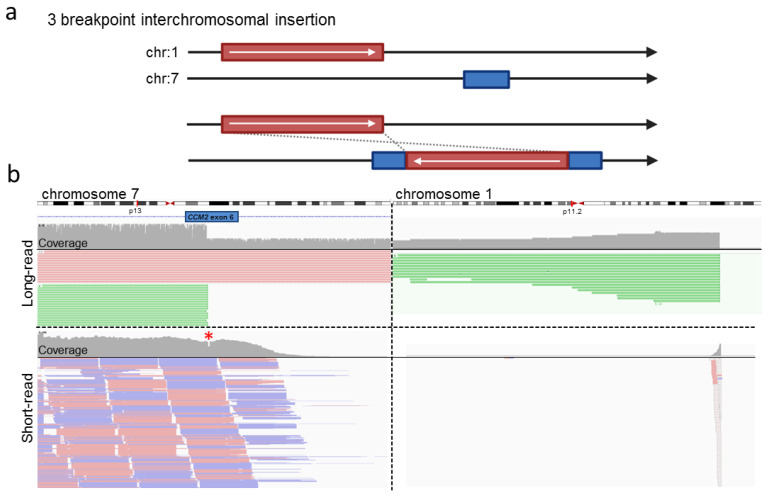
Nanopore sequencing confidently detected an interchromosomal insertion in *CCM2*. (**a**) Schematic representation of the interchromosomal insertion in *CCM2*. (**b**) Long-read sequencing data of a previously described heterozygous interchromosomal insertion revealed about 50% of reads covering *CCM2* terminate in exon 6 (green). These reads all had a supplementary alignment mapping to chromosome 1. Short-read data showed consistent coverage of *CCM2* exon 6. The coverage at the position of the variant breakpoints was reduced (*, red star), and a limited number of reads had a supplementary alignment on chromosome 1. Most reads bridging to chromosome 1 had a mapping quality equal to 0 (hollow reads). Read data were inspected in IGV [23].

In the next step, we re-analyzed a known heterozygous interchromosomal insertion of chromosome 1 material into the coding region of *CCM2* (Figure 5a). This variant which we had previously called from short read gene panel sequencing data with the SureCall software, was an ideal positive control for our approach because it already had been extensively characterized and confirmed by FISH [9]. Long-read sequencing of DNA with this variant (Table S3) generated single reads longer than 20 kb. These reads spanned the breakpoint located in *CCM2* exon 6 with subsequent mapping of bases to chromosome 1 (Figure 5b). Due to the long read length, confident mapping of supplementary alignments on 1p11.2 was possible. As there were no reads covering the entire 294 kb insertion in our oxford nanopore data, an additional crRNA with a binding site downstream of the breakpoint in *CCM2* would have been required in a diagnostic context to identify the second breakpoint on chromosome 1. However, due to the limited availability of DNA from this positive control, additional sequencing was unfortunately not possible in our current study.

Taken together, our data show that even complex SVs can be detected with Cas9-based oxford nanopore sequencing.

### 2.4. Targeted Nanopore Sequencing on the Flongle Flow Cell Is Also Able to Determine Variant Breakpoints 

**Figure 6:**
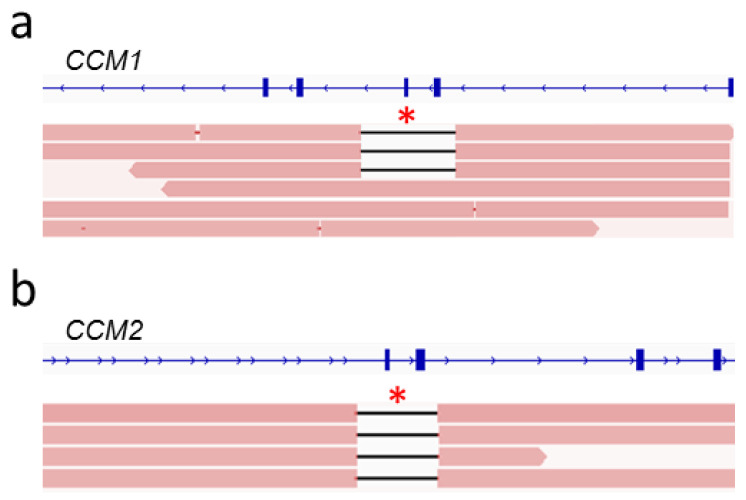
Sequencing on Flongle flow cells detected variant breakpoints in a shallow Cas9-mediated long-read approach. (**a**,**b**) Sequencing data from two dual-cut excision approaches sequenced on a Flongle flow cell. (**a**) Detection of the two-exon deletion (*, red star) of exons 12 and 13 in *CCM1* (Figure 1) with a sequencing coverage of 6×. (**b**) Re-sequencing of a heterozygous two-exon deletion (*, red star) of exons 3 and 4 in *CCM2* with a Flongle flow cell yielded a sequencing coverage of 4×. No wild-type reads were generated. Read data were inspected in IGV [23]. The Locus Reference Genomic (LRG) transcripts are shown (**a**,**b**).

To reduce the sequencing costs, we next tested whether CNV breakpoints could also be refined by Cas9-mediated sequencing on Flongle flow cells. We first used a dual-cut approach with two crRNAs flanking the breakpoints of the intragenic *CCM1* deletion described in Figure 1 and a second intragenic deletion in *CCM2*. Using high molecular weight DNA or medium-sized DNA treated with the Short Read Eliminator Kit, respectively, the deletion of exons 12 and 13 in *CCM1* (Figure 6a) and the deletion of exons 4 and 5 in *CCM2* (Figure 6b) could be re-identified with Flongle sequencing. However, the extreme bias in the variant allele read frequency of the second sample indicated limited reliability of this approach. We also tested a single-cut approach for the large *CCM1* deletion described in Figure 2. Although we were able to detect the deletion, only one read covered the target region (Figure S1). Although a dual-cut approach appears to provide higher coverage, Flongle sequencing is significantly less reliable than Cas9-mediated sequencing on standard MinION flow cells. 

## 3. Discussion

In this study, we demonstrate that the implementation of Cas9-mediated long-read sequencing can significantly improve the sensitivity and accuracy of molecular genetic diagnostics for CCM patients when used as a complementary analytical approach. It allows not only the specification of CNV breakpoints but also the detection of complex germline SVs that can be easily missed by targeted NGS approaches. 

Large deletions have been reported for all three *CCM* genes [2,24,25,26,27,28,29,30,31,32]. Their size ranges from a few hundred kilobases to 1.9 megabases [24,30,31]. However, the actual size of CNVs in *CCM1*, *CCM2*, or *CCM3* is often unknown because the identification methods do not allow accurate mapping of the breakpoints [24,26,31,33]. Indeed, we show here that the deletion of the first six exons of *CCM1* was much larger than initially thought and also included parts of the neighboring *ANKIB1* gene. Other studies also identified *CCM1* gene deletions with a breakpoint in *ANKIB1* [30]. Variants involving neighboring genes are also known for *CCM2* [4,25,33] and *CCM3* [28]. 

In molecular diagnostics, a variety of wet lab assays can be used to screen for CNVs. The MLPA technique, for example, is widely used to detect large deletions and duplications in many well-known disease genes. The resolution depends on the design of the MLPA probes, and non-coding exons might not be completely covered. Microarray-based high-density comparative genomic hybridization (aCGH) offers high-throughput analyses with a minimum resolution of 500 bp [34]. However, aCGH requires special equipment and is limited in detecting small duplications [35]. Bioinformatic advances have also made it possible to detect CNVs from high-throughput short-read panel sequencing data, allowing parallel detection of SNVs, indels, and CNVs with exon-level resolution [3,36]. Nevertheless, all techniques have disadvantages regarding targeted analyses for a familial CNV. Short-read panel sequencing is rarely used to detect known familial CNVs due to its rather high costs. However, even MLPA analysis can become time-consuming and expensive if parallel analysis of multiple samples is impossible [36]. Especially in large CCM families, several relatives often need to be screened for a familial CNV at different time points [37]. For example, in one family described here (Figure 2), nine members were sequentially tested for the identified *CCM1* deletion by MLPA. Long-read sequencing is a promising option in this context. Once the breakpoints of a familial CNV have been established by long-read sequencing, subsequent analyses could be performed with inexpensive allele-specific PCR. For predictive analyses, this can reduce the hands-on time to a few hours while keeping reagent costs very low. Moreover, the MinION platform used in this study has low initial investment costs [38]. The relatively high costs of standard MinION flow cells, however, still represent a limitation of this approach. Although we could also refine the breakpoints of known CNVs with sequencing on the much cheaper Flongle flow cell, this method is not yet reproducible enough for diagnostic use. Nonetheless, since long-read sequencing with a standard MinION flow cell would be performed only once in the index patient, it can still be a cost-effective option when the need for further analyses of family members is foreseeable.

The efficiencies of Cas9-mediated target selection and Oxford Nanopore sequencing reported in the literature vary within a wide range. Depending on the size of the respective target region and the individual experimental approach, on-target coverages of >500× but also <30× have been described [39,40,41,42,43,44,45]. Especially when large genes are to be covered in a Cas9-based approach with long reads, the achievable sequencing depth often becomes an issue [41]. The sequencing depths achieved in our study, which were ≥30× in all but one case (Tables S2 and S3), are within the expected range. While a higher on-target coverage would be desirable for screening approaches, these sequencing depths are more than sufficient for confirmatory analyses of known CNVs.

Breakpoint specification of known CNVs by long-read sequencing not only enables the development of efficient PCR-based assays for familial analyses but can also provide insight into the origin of CNVs. Mechanisms leading to CNV formation often involve the recombination of homologous DNA sequences. Alu-repeats, for example, belong to the short interspersed nuclear elements (SINES) that largely facilitate structural variation in the genome. They are characterized as repetitive regions about 300 bp in size and contribute to almost 11% of the human genome [46]. Because of their high homology, Alu-repeats are responsible for genomic instability. Following a double-strand break, non-allelic homologous recombination mediated by Alu-repeats can lead to deletions, duplications, and complex SVs [46]. Alu-mediated CNVs and SVs have been found in various disorders, like chronic granulomatous disease [47], limb-girdle muscular dystrophy 5 [48], and glaucoma [49]. In CCM, Alu-repeat recombination is also a known disease mechanism. Liquori and colleagues discovered a founder mutation resulting from recombination in *CCM2* between an AluSx- and an AluSg-repeat region which led to the deletion of exons 2 to 10 [4]. In our second familial case, breakpoint specification with long-read sequencing also revealed Alu-mediated recombination as a possible cause of the identified deletion (Figure 3). Variant breakpoints were located in an AluSq2- and an AluSg-repeat, respectively. 

Beyond accurate characterization of variant breakpoints, the utility of long-read sequencing as a complementary analysis to gene panel sequencing is also demonstrated by its ability to detect more complex SVs that are usually not captured by targeted short-read sequencing approaches [50]. Inversions or translocations, for example, might easily be missed when breakpoints are not located in coding regions. Unfortunately, short-read WGS, which has higher sensitivity for complex SVs, is not yet an option for routine diagnosis of monogenic diseases with well-established risk genes due to high investment costs and bioinformatics requirements. Targeted long-read sequencing, on the other hand, is relatively easy to implement and might confidently close the diagnostic gap for SVs [18]. Consistent with the experience of other groups in resolving complex structural rearrangements, our targeted long-read sequencing assay reliably re-identified an interchromosomal insertion and a copy number-neutral inversion in the *CCM2* gene that had previously been detected in short-read approaches and elaborately validated [8,9]. However, it should be emphasized that a diagnostic SV screening approach would require higher bioinformatic skills and the use of more variant callers than an approach to refine the breakpoints of known CNVs. Despite continuous advances in sequencing chemistry and computational compensation [51,52,53], nanopore sequencing is also not yet suitable as a stand-alone diagnostic approach. In particular, the relatively high error rate of nanopore sequencing and the risk of missing whole gene deletions in Cas9-mediated targeted long-read sequencing currently limit its diagnostic power. While short-read panel sequencing remains the most cost-effective method for detecting SNVs, indels, and CNVs as a first-line approach, complementary nanopore sequencing can reduce costs and turnaround time for pathogenic familial CNVs and may also be used as a second-line approach to screen for complex SVs. 

Taken together, we demonstrate that the implementation of long-read sequencing can be a great benefit to CCM diagnostics. Our experience also suggests that this approach can very easily be transferred to other diagnostic questions.

## 4. Materials and Methods

### 4.1. Design of crRNAs

CrRNAs for *CCM1*, *CCM2*, and *CCM3* (Table S1) were designed and checked with the crRNA design tool from Integrated DNA Technologies (accessed between 5 May 2022 and 1 July 2022, Integrated DNA Technologies, Coralville, IA, USA). If possible, crRNAs with binding sites in exonic regions were used. If an intronic crRNA location was inevitable, we used crRNAs with binding sites outside of repetitive or poorly characterized regions. It was ensured that crRNA binding sites did not contain SNVs with a minor allele frequency greater than 0.01% listed in gnomAD v2.1.1 (accessed between 5 May 2022 and 1 July 2022). 

### 4.2. Nanopore Sequencing 

All study participants gave written informed consent for genetic analyses. High-molecular-weight genomic DNA was isolated from fresh blood or frozen blood samples using the Monarch HMW DNA Extraction Kit (New England BioLabs, Ipswich, MA, USA). The NucleoSpin Blood L Kit (Macherey-Nagel, Düren, Germany) was used to isolate medium-sized DNA fragments that are typically used in routine diagnostics. To eliminate short reads, the Short Read Eliminator Kit (Circulomics, Baltimore, MD, USA) was used. If necessary, additional purification of genomic DNA samples was performed by magnetic bead clean-up with AMPure XP beads (Beckman Coulter, Brea, CA, USA). Target selection was performed either with the Cas9 Sequencing Kit (SQK-CS9109, Oxford Nanopore Technologies, Oxford, United Kingdom) or the Ligation Sequencing Kit (SQK-CS9109, Oxford Nanopore Technologies) in combination with the following additional components: dATP Solution, NEBNext Quick Ligation Module, Quick CIP, Taq DNA Polymerase (New England BioLabs) and IDTE pH 8.0 (Integrated DNA Technologies). For Cas9 cleavage, Alt-R S.p. Cas9 Nuclease V3 (Integrated DNA Technologies), Alt-R CRISPR-Cas9 tracrRNA (Integrated DNA Technologies), and Alt-R CRISPR-Cas9 crRNAs (Integrated DNA Technologies) were used. Briefly, for targeted sequencing of a known variant, up to 5 µg of genomic DNA was used for dephosphorylation. DNA was then cleaved by RNP complexes with crRNAs located directly up- or downstream of the variant. After poly-A-tailing and AMPure XP bead purification, sequencing adapters were ligated, and sequencing was started. Long-read-sequencing was performed on a MinION device (Oxford Nanopore Technologies) equipped with R9.4.1 flow cells (Oxford Nanopore Technologies). 

For combined sequencing of *CCM1*, *CCM2*, and *CCM3*, up to 15 µg of genomic DNA were divided and dephosphorylated in three separate tubes. The tubes were treated with RNP complexes originating from one of three crRNA-pools (Table S1). After poly-A-tailing, the three tubes were mixed, and AMPure XP bead purification, adapter ligation, and sequencing were performed as usual. Target selection for Cas9-mediated nanopore sequencing on Flongle flow cells (Oxford Nanopore Technologies) was performed with the Cas9 Sequencing Kit as mentioned above. Loading of the Flongle flow cell was done with the Flongle Flow Cell Priming Kit (EXP-FSE001, Oxford Nanopore Technologies) and according to the “Loading the Flongle flow cell” section of the SQK-LSK109 sequencing protocol.

Live basecalling was performed with Guppy integrated in the MinKNOW software (version 22.03.6, Oxford Nanopore Technologies). FASTQ files were combined with cygwin64 (version 1.7.35), and alignment to the GRCh37 reference genome was carried out with the MinKNOW software (version 22.03.6, Oxford Nanopore Technologies) utilizing the minimap2 aligner. Index files were generated with samtools (version 1.10) for bioinformatics analyses and igvtools [23] for visual inspection. SVs or CNVs were called with cuteSV (version 2.0.2) [54] and the human variation workflow of the EPI2ME Labs software (version 3.1.5, Oxford Nanopore Technologies) which utilizes Sniffels2 [55]. After narrowing down the SV or CNV breakpoints with the bioinformatic tools, long-read sequencing data were further visually inspected with IGV [23] (version 2.13.0, Broad Institute, Cambridge, MA, USA) to design variant-specific PCR assays.

### 4.3. Multiplex Ligation-Dependent Probe Amplification, PCR, and Sanger Sequencing

For copy number variation analysis by MLPA, the kits P130-A3 and P131-B1 containing probes for *CCM1*, *CCM2*, and *CCM3* were used according to the manufacturer’s instructions (MRC-Holland, Amsterdam, The Netherlands). Fragment analysis was performed on a SeqStudio Genetic Analyzer (Applied Biosystems, Waltham, MA, USA) or an ABI 310 Genetic Analyzer (Applied Biosystems), and the data were analyzed with the SeqPilot software (version 5.1.0, JSI Medical Systems, Ettenheim, Germany). Visualization was performed with the GraphPad Prism software (version 8.0.1, GraphPad Software, Inc, San Diego, CA, USA). 

For breakpoint confirmation, variant-specific primers (Integrated DNA Technologies) were designed, and PCR reactions were carried out with the Taq DNA-Polymerase (Thermo Fisher Scientific, Waltham, MA, USA). To amplify GC-rich regions, the OneTaq DNA Polymerase (New England BioLabs) or the GC-RICH PCR-System (Roche, Basel, Switzerland) was used with GC-Rich Buffer. PCR products were evaluated on a 1.5% agarose gel imaged on a ChemiDoc XRS+ system (BioRad, Hercules, CA, USA). Bands were cut from the gel and extracted with the Zymoclean Gel DNA Recovery Kit (Zymo Research, Irvine, CA, USA). The sequencing reaction was done with the BigDye Terminator v3.1 Cycle Sequencing Kit (Thermo Fisher Scientific). After cleanup, the samples were sequenced on a SeqStudio Genetic Analyzer. Sequencing data were evaluated with SnapGene Viewer (Dotmaticsm, Boston, MA, USA).

### 4.4. Illumina Sequencing

Hybridization capture-based target enrichment of genomic DNA samples for *CCM1*, *CCM2*, and *CCM3* was performed using an Agilent SureSelect^QXT^ custom enrichment kit (Panel ID: 3152261, Agilent Technologies, Santa Clara, CA, USA). Illumina sequencing was performed on an Illumina MiSeq platform with 2 × 150 cycles (Illumina, San Diego, CA, USA). Basecalling and alignment to the GRCh37 reference genome were performed with the MiSeq Reporter Software (version 2.6.2, Illumina). Data were inspected with IGV [23] (version 2.13.0).

## Data Availability

The data presented in this study are available on request from the corresponding author. The raw sequencing data are not publicly available because they contain additional genetic information that could compromise the privacy of research participants.

## Supplementary Materials

The following supporting information can be downloaded at: https://www.mdpi.com/article/10.3390/ijms232415639/s1.
