# Supplementary material for: Cas9-Mediated Nanopore Sequencing Enables Precise Characterization of Structural Variants in CCM Genes
Source: Int J Mol Sci. 2022 Dec 9;23(24):15639. doi: 10.3390/ijms232415639 (PMC9779250; doi:10.3390/ijms232415639)
Supplement: Supplementary file 1 [file ijms-23-15639-s001.zip › Skowronek_et_al._Supplementary Material.pdf]

# Supplementary Material

**Table S1.** Sequencing reactions for SV screening in CCM and crRNAs.

| Reaction | Gene | No. | Name | crRNA sequence | Used in targeted sequencing |
|---|---|---|---|---|---|
| 1 | *CCM1* | I | CD.Cas9.RFSH5951.AA | ATTCTCGGTGTAGTGGTCAA | *CCM2* 24kb inversion (Figure 4) |
| | | IV | CD.Cas9.KHSK6404.AA | GGTTAGACAATAATCTTGAT | *CCM1* exon 12 – 13 deletion (Figure 6a) |
| | *CCM2* | I | CD.Cas9.CLYJ9692.AD | GTGCCCCTAACCAATCACTT | |
| | | IIX | CD.Cas9.NZXJ8292.AC | GGTGGGATAATCAACTACTA | *CCM2* 24kb inversion (Figure 4) |
| | | V | CD.Cas9.HRHZ5617.AQ | TTGCAGGTCGACTCCCACGC | *CCM2* exon 4 – 5 deletion (Figure 6b) |
| | *CCM3* | III | CD.Cas9.QVBM8621.AD | TCTAAGTGGGAGAACTCCCC | |
| 2 | *CCM1* | II | Hs.Cas9.KRIT1.1.AA | ACCAGTTAGATAGTGACCAC | *CCM1* exon 12 – 13 deletion (Figure 6a) |
| | *CCM2* | III | CD.Cas9.GVVJ4790.AF | TCGTATGAGGCAGGTACCAT | |
| | | VII | CD.Cas9.JXFD3882.AA | GCAAGGCCATGTTCAACCAG | *CCM2* exon 4 – 5 deletion (Figure 6b) |
| | *CCM3* | I | CD.Cas9.SFRX5440.AF | TCCAATATTCCAGAGCCACT | |
| | | V | CD.Cas9.BVPB2717.AL | CCACCTCGTCCTCAATTAGC | |
| | | VI | CD.Cas9.MRWC3202.AA | AATGCTATAAACGACCAAGG | |
| 3 | *CCM1* | III | CD.Cas9.KDPJ4588.AA | TGACTAATCTGGATCCTCAA | |
| | *CCM2* | IV | CD.Cas9.VXFG0396.AA | TGGGTTGAAACGTTTGGCAA | |
| | | II | CD.Cas9.HWXB2549.AB | TAACTCTGTTCTATTGCTAC | |
| | | VI | Hs.Cas9.CCM2.1.AB | GGTCAGTTAACGTCCATACC | *CCM2* exon 6 – 11 deletion (Figure 3); *CCM2* interchromosomal insertion (Figure 5) |
| | *CCM3* | II | CD.Cas9.JJYJ9047.AL | ATAAGTACTCCGCTCTTTGA | |
| | | IV | Hs.Cas9.PDCD10.1.AA | CACGGAGTCCCTTCTTCGTA | |

For targeted sequencing of the familial *CCM1* exon 2 – 6 deletion (Figure 2), a crRNA not included in the CCM panel was used: Hs.Cas9.KRIT1.1.AC (GGAGCTCCTAGACCAAAGTA).

**Table S2. Run summary for Cas9-mediated nanopore sequencing of *CCM* genes using the crRNA panel.** Values for the sequencing run shown in Figure 1 are depicted.

| | **Target region size [bp]** | **On target reads** | **Mean target coverage** | **Total mapped reads** | **Called bases [Gb]** | **Theoretical genome coverage*** | **Enrichment ** ** |
|---|---|---|---|---|---|---|---|
| *CCM1* | 47,131 | 256 (0.04 %) | 89.5x (SD = 19.6) | 608,563 | 3.03 | 0.97x | 92-fold |
| *CCM2* | 76,282 | 396 (0.06 %) | 71.9x (SD = 25.3) | | | | 74-fold |
| *CCM3* | 51,594 | 147 (0.02 %) | 31.0x (SD = 18.9) | | | | 32-fold |

*Ratio: (called bases)/(reference genome size [3.137 Gb])

**Ratio: (mean target coverage)/(theoretical genome coverage)

**Table S3. Run summary for Cas9-mediated nanopore sequencing in targeted approaches.** Values for the sequencing runs shown in Figure 2-5 are depicted.

| | ***CCM1* large deletion** (Figure 2) | | ***CCM2* large deletion** (Figure 3) | | ***CCM2* inversion** (Figure 4) | | ***CCM2* interchromosomal insertion** (Figure 5) |
|---|---|---|---|---|---|---|---|
| Number of crRNAs used | 1 | | 1 | | 2 | | 1 |
| Called bases [Gb] | 0.09 | | 1.51 | | 0.56 | | 0.57 |
| Theoretical genome coverage* | 0.03x | | 0.48x | | 0.18x | | 0.18x |
| BP-spanning coverage | BP1: 12x | BP2: 8x | BP1: 104x | BP2: 58x | BP1: 55x | BP2: 41x | 30x |
| BP-spanning enrichment** | 400-fold | 267-fold | 217-fold | 121-fold | 306-fold | 228-fold | 167-fold |

BP: breakpoint
*Ratio: (called bases)/(reference genome size [3.137 Gb])
**Ratio: (BP-spanning coverage)/(theoretical genome coverage)

**Table S4.** Bioinformatic evaluation to narrow down breakpoints of CNVs or SVs.

| Gene | Variant | Genomic location (Sniffels2) | Genomic location (cuteSV) |
|---|---|---|---|
| CNVs | | | |
| *CCM1* | Exon 12 – 13 deletion (Figure 1) | chr7:91853863 - 91856415 | chr7:91853863 - 91856415 |
| *CCM1* | Exon 2 – 6 deletion (Figure 2) | chr7:91868272 - 91878609 | chr7:91868270 – 91878607 |
| *CCM2* | Exon 6 – 11 deletion (Figure 3) | chr7:45107067 - 45130811 | chr7:45107051 - 45130953 |
| SVs | | | |
| *CCM2* | Inversion (Figure 4) | n.d.* | chr7:45031566 - 45055822 |
| *CCM2* | Interchromosomal insertion (Figure 5) | n.d.* | chr7:45108096 |

* not detected.

**Table S5.** PCR primers for breakpoint confirmation and familial analyses.

| Gene | Variant | Forward Primer (5′-3′) | Reverse Primer (5′-3′) |
|---|---|---|---|
| *CCM1* | Exon 12 – 13 deletion (Figure 1) | GGTTAATGCCTGGAGAGGCT | GCATGGACAAACATTACCATACT |
| *CCM1* | Exon 2 – 6 deletion (Figure 2) | TGCCTTCTGTGGGCAGGTAA | AAACAACCTTGCAACAGGGTC |
| *CCM2* | Exon 6 – 11 deletion (Figure 3) | TGGCTTAGAAGGCTGGGTTG | GCCTGGCTTGCTGACCTTTC |

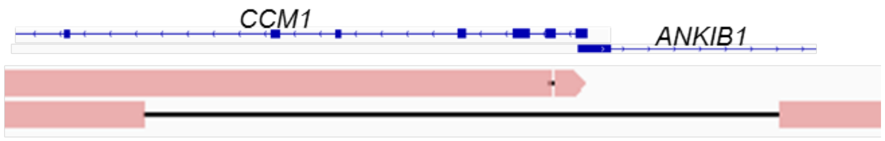


**Figure S1.** Sequencing of the familial *CCM1* exon 2 – 6 deletion (Figure 2) on a Flongle flow cell was able to detect the variant breakpoints. The variant was covered by one sequencing read. A single-cut approach was used. Read data was inspected in IGV [23]. The Locus Reference Genomic (LRG) transcript (*CCM1*) and RefSeq transcript (*ANKIB1*) are shown.
